# Exploring Nutritional Status and Metabolic Imbalances in Children with FASD: A Cross-Sectional Study

**DOI:** 10.3390/nu16193401

**Published:** 2024-10-07

**Authors:** Katarzyna Anna Dylag, Wiktoria Wieczorek-Stawinska, Katarzyna Burkot, Lukasz Drzewiecki, Katarzyna Przybyszewska, Aleksandra Tokarz, Paulina Dumnicka

**Affiliations:** 1 Department of Pathophysiology, Jagiellonian University Medical College, 31-121 Krakow, Poland; 2 St. Louis Children Hospital, 31-503 Krakow, Polanda.tokarz@dzieciecyszpital.pl (A.T.); 3 Chair of Medical Biochemistry, Jagiellonian University Medical College, 31-034 Krakow, Poland

**Keywords:** foetal alcohol spectrum disorder, FASD, prenatal alcohol exposure, PAE, malnutrition, macronutrient, micronutrient, nutrimetabolomics, paediatric

## Abstract

Background/Objectives: Malnutrition is a significant concern in paediatric populations, particularly among children with neurodevelopmental disorders such as foetal alcohol spectrum disorder (FASD). This study aimed to examine macronutrient and micronutrient imbalances and assess the nutritional status of a group of patients with FASD. Methods: This study involved an analysis of the serum levels of key nutrients in a group of children diagnosed with FASD. Macronutrients and micronutrients were measured to identify any imbalances, including vitamin D, B12, E, A, albumin, and serum protein, among others. Results: The study found a high prevalence of vitamin D deficiency among the patients. Additionally, elevated serum concentrations of micronutrients such as vitamin B12, E, and A were observed in 8%, 7%, and 19% of patients, respectively. Macronutrient imbalances were noted, including high levels of albumin and serum protein, indicating a possible metabolic disturbance. Unexpectedly, high rates of hypercholesterolemia were observed, raising concerns about an increased risk of metabolic syndrome in this population. Conclusions: These findings suggest that the principal issue among patients with FASD is an altered metabolism rather than nutritional deficiencies. Potential causes of these abnormalities could include oxidative stress and changes in body composition. The results underline the need for further research to better understand the unique nutritional challenges in children with FASD and to guide the development of targeted therapeutic strategies.

## 1. Introduction

Foetal alcohol spectrum disorder (FASD) is an umbrella term for a group of conditions resulting from prenatal alcohol exposure (PAE). FASD is considered one of the leading causes of intellectual disability in economically advanced countries [1]. The prevalence of FASD is estimated at 7.7–22.7 cases per 1000 individuals worldwide [2,3]. As a spectrum disorder, FASD can manifest with a variety of symptoms; however, neurodevelopmental problems occur among all patients. Patients with FASD experience challenges in both cognitive and psychosocial functioning. Thus, much of the previous research on FASD has focused on the neurological and neuropsychological aspects [4,5]. Such an approach has often overlooked the physical health challenges faced by patients with FASD.

There is an unambiguous relationship between childhood nutrition, neurodevelopment, and physical health [6]. Until now, several studies have investigated nutritional issues among patients with FASD. An early example of research in the field includes the report of van Dyke et al. [7], who indicated feeding dysfunction among children diagnosed with foetal alcohol syndrome (FAS). Researchers observed a high frequency of feeding disorders among infants with prenatal alcohol exposure. The problems diagnosed included motor dysfunction with a limited suck pattern and fatigability [7]. Researchers have also shown that feeding problems in this group do not fade after infancy. Patients with FASD were shown to experience delayed acquisition of self-feeding behaviours and introduction of solid food [7] and impaired self-regulation of hunger and satiety [8,9]. In this group, a tendency to snack and hide food was also reported [9]. To date, the actual nutritional status of children with FASD has been researched in only a few studies. Nguyen et al. performed an analysis of the daily macronutrient and micronutrient intake of patients with FASD and reported that their diet is deficient in fibre, potassium, vitamins E, K, omega-3 fatty acids, and choline [10]. Fuglestad et al. came to similar conclusions; however, they noted that vitamin D and calcium deficiencies are also significant [11]. To date, research has tended to focus on inadequate intake of micronutrients and macronutrients rather than actual deficiencies. The objective of this study is to analyse macronutrient and micronutrient imbalances in a group of patients with FASD and to evaluate their nutritional status using objective laboratory measurements.

## 2. Materials and Methods

### 2.1. Study Design and Participants

This study was carried out as a prospective observational study in the years 2022–2024 and included patients of the FASD Diagnostic Centre of St. Louis Children’s Hospital in Kraków, Poland.

The study group consisted of 75 patients with confirmed diagnosis of FASD. The patients were offered participation in the study by an independent researcher. The inclusion criteria were the following: diagnosis of FASD established by a multidisciplinary team according to Polish diagnostic criteria [12] and an age between 9 months and 17 years at the time of enrolment. Exclusion criteria were comorbid gastrointestinal conditions that could result in nutritional malabsorption treatment with enteral or parenteral nutrition, using dietary supplements containing mictronutrients other than infant formula, or an acute illness that could affect laboratory tests results at the time of enrolment. One patient met the exclusion criteria.

Information on the type of custody and personal information was obtained from the patients in a medical interview. Anthropometric measurements were taken by a qualified nurse on a calibrated paediatric scale and stadiometer according to the NHANES manual [13]. Anthropometric values (weight, height, and body mass index, BMI) were compared with the Polish percentile growth charts and assigned to the respective category or channel (below 3rd, 3rd, above 3rd and below 10th, 10th, above 10th and below 25th, 25th, above 25th and below 50th, 50th, above 50th and below 75th, 75th, above 75th and below 90th, 90th, above 90th and below 97th, 97th, or above 97th percentile) [14]. Blood samples for laboratory measurements were collected in the morning hours after overnight fasting. A qualified paediatric nurse took the blood sample and local anaesthesia for blood withdrawal was provided on parental request.

### 2.2. Ethics

The study protocol was approved by the regional ethics committee of the Regional Board of Physicians (no: 50/KBL/OIL/2022, date: 11 April 2022). The researchers obtained written informed consent from the parent/caregiver. Separate consent was obtained from children over 13 years of age. All children, including those under 13 years of age, were informed about the study and all their questions about the study were answered.

### 2.3. Laboratory Methods

For haematological measurements, blood was collected in a K_3_EDTA tube (Vacuette, Greiner Bio-One, Kremsmünster, Austria) and analysed within 3 h of collection. The complete blood count was performed using the Sysmex XN 550 analyser (Sysmex Corporation, Kobe, Japan). For ionised calcium measurements, blood samples were collected in heparinised capillary plastic tubes (Radiometer, Copenhagen, Denmark) and analysed within 15 min of collection with the ABL 90 Flex Plus analyser (Radiometer, Copenhagen, Denmark). Furthermore, three serum samples were collected in serum tubes with a cloth activator and serum separator (Vacuette, Greiner Bio-One, Kremsmünster, Austria). These samples were centrifuged at 3800 rpm for 10 min at 4 °C within 30 min after collection. Measurements of total protein, albumin, total cholesterol, ferritin, parathormone, phosphate, magnesium, and total calcium were performed within 3 h of blood collection using the Architect ci4100 analyser (Abbott Laboratories, Chicago, IL, US). Two other serum samples were frozen at −20 °C for further analysis. Vitamin B12 and prealbumin were measured using the Alinity analyser (Abbott Laboratories, Chicago, IL, US) and vitamin D using the Liaison analyser (DiaSorin, Sallugia, VC, Italy). Vitamin A and vitamin E were measured with the LC-2030C DAD HPLC system (Shimadzu, Tokyo, Japan). The laboratory methods and the reference intervals used in the study are summarised in Table 1.

### 2.4. Statistical Analysis

For categorical variables, the number of patients and percentage of the group were reported. The contingency tables were analysed using Pearson’s chi-square test. The quantitative variables were checked for normality using Shapiro–Wilk’s test. Arithmetic mean ± standard deviation was reported for normally distributed variables and median (lower; upper quartile) for those non-normally distributed. The *t*-test and the Mann–Whitney test was used for subgroup comparisons, respectively. Additionally, we analysed the differences between the subgroups in logistic regression adjusted to sex and age. Spearman rank correlation coefficient was used to study the association between weight, height, and BMI percentile categories (ordered variables) and the results of laboratory tests. The Pearson correlation coefficient was used to assess correlations between normally distributed quantitative variables. All the tests were two-tailed; *p* < 0.05 indicated statistically significant results. Statistica 13.3 software (TIBCO Software Inc., Tulsa, OK, US) was used for calculations.

## 3. Results

### 3.1. Sample Characteristics

**Figure 1:**
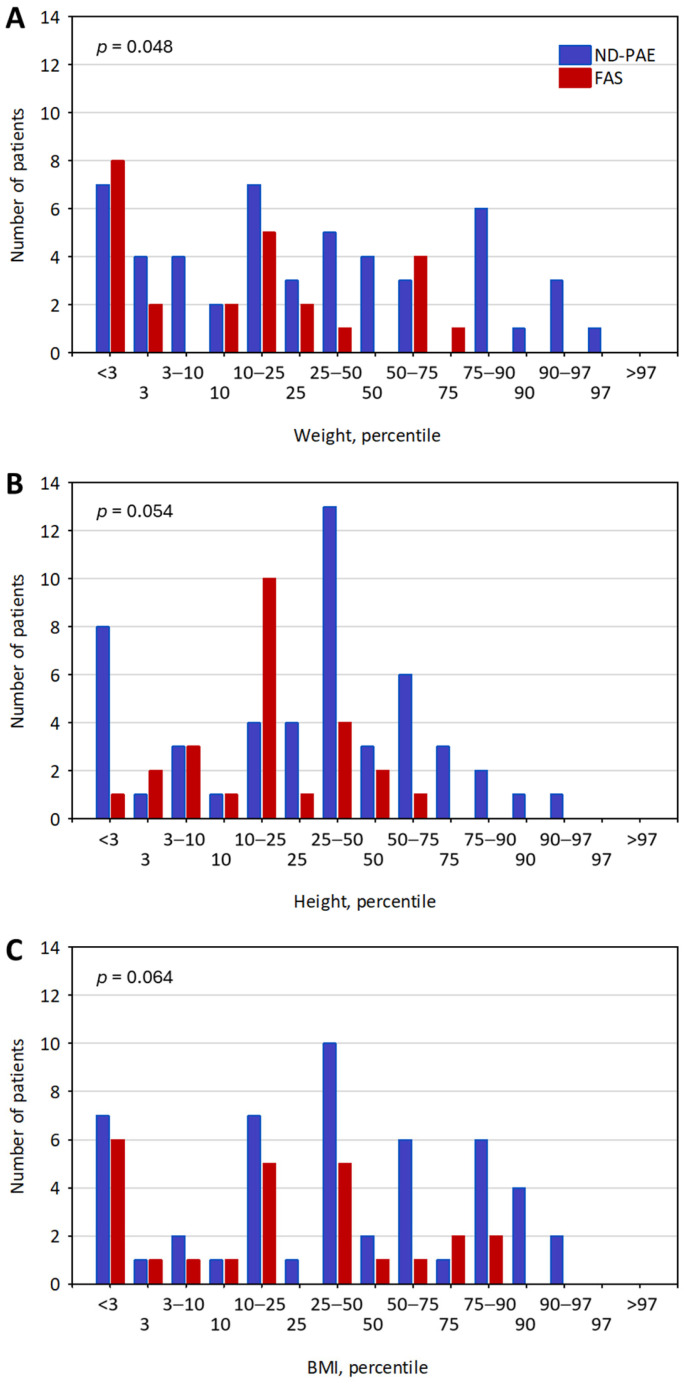
Histograms showing the number of children with FAS (red) and ND-PAE (blue) in each percentile category for weight (**A**), height (**B**), and BMI (**C**). The *p*-values for the difference between the FAS and ND-PAE groups are shown in the graphs. The category depicted “3–10” denotes children in the channel of above 3rd and below 10th percentile; the same rule applies for further categories. The “<” and “>” signs in those categories were omitted to enable readability of the *x*-axis captures.

The studied group included patients aged 1 to 16 years, the median age was 7 years (3; 9). The group consisted of 25 patients with a diagnosis of foetal alcohol syndrome (FAS) and 50 patients diagnosed with neurodevelopmental problems with prenatal alcohol exposure (ND-PAE). In total, 3 children were in institutional care, 34 were in foster care, 34 were raised in adoptive families, and 5 remained with their biological parents. The demographic characteristics of the patients are presented in Table 1. The anthropometric characteristics of the sample are presented in Figure 1.

### 3.2. Laboratory Test Results

The results of the average laboratory tests of the children studied are presented in Table 2. There were no statistically significant differences between the subgroups diagnosed with FAS and ND-PAE (Table 2). Consistently, we did not observe such differences in the logistic regression adjusted to sex and age.

**Figure 2:**
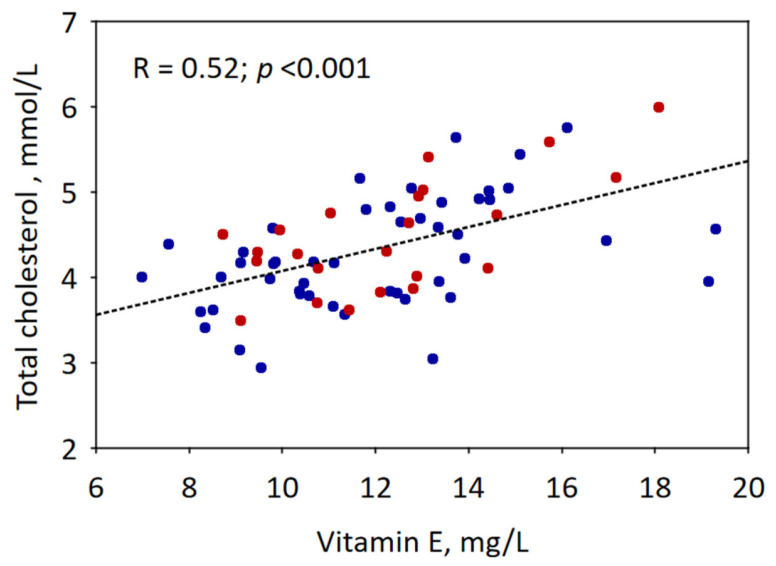
Association between vitamin E and total cholesterol in the studied group of children with FASD. The dotted line denotes the linear regression line and R denotes the Pearson correlation coefficient for the entire group. The results for children with FAS are shown in red and those of ND-PAE are shown in blue.

In our sample, we identified biomarkers of malnutrition in relatively few patients. A decrease in ferritin was observed in 5% of the patients, a vitamin D deficiency in 35% of the patients, hypophosphatemia in 5% of the patients, decreased zinc levels in 1% of the patients, and a vitamin A deficiency in 3% of the patients (Table 3). Interestingly, increased concentrations of vitamin B12 and vitamin A and E were observed, respectively, in 8%, 7%, and 19% of patients (Table 3). Furthermore, hyperalbuminemia and hyperproteinaemia were identified in 17% and 31% of the patients, while none of the participants had decreased prealbumin levels (Table 3). Hypercholesterolemia was observed in 17% of the patients (Table 3). Hypervitaminosis E was positively correlated with total cholesterol levels (Figure 2). Increased zinc levels were observed among 19% of the subjects (Table 3).

We observed a significant positive correlation between the weight percentile of the subjects and the prealbumin concentration, the B12 concentration, the vitamin D concentration, and the haemoglobin concentration (Table 4). The BMI percentile was positively correlated with vitamin B12 concentration and zinc concentration (Table 4). Negative correlations were observed for white blood cells, neutrophils and lymphocyte count and weight percentile and zinc and height percentile, as well as white blood cell lymphocyte levels and BMI percentile (Table 4). Neither vitamin B12 nor ferritin concentrations were significantly correlated with red blood cell count, haemoglobin, or haematocrit (*p* > 0.1 for all correlations).

Children in institutional or foster care were significantly younger than those in adoptive families (5.76 ± 3.77 vs. 8.03 ± 3.77; *p* = 0.014). There were no differences between these groups in the percentiles of height (*p* = 0.37), weight (*p* = 0.65), or BMI (*p* = 0.48). Institutional or foster care was more commonly associated with increased (i.e., above the upper reference limit) serum albumin and increased zinc (Table 5). The results of other laboratory tests did not differ significantly between the groups.

Finally, we compared the group of eleven youngest children (up to 2 years of age) with the older ones. High albumin was significantly more common in children up to 2 years of age compared to older children (Table 5). Furthermore, the youngest children were characterised by significantly lower weight percentiles (*p* = 0.045), while there were no statistically significant differences in height or BMI percentiles.

We did not observe any significant differences in the laboratory test results between girls and boys.

## 4. Discussion

Malnutrition in the paediatric age group is a major public health problem [22]. Although most cases worldwide are caused by food insecurity, being diagnosed with a developmental disability is considered a risk factor for malnutrition [23]. There are various methods to assess the nutritional status of children, with anthropometric parameters such as percentile curves being the first [24] and nutritional indices to follow [25]. However, in special populations such as children with FASD, in which growth deficiencies can be considered a part of the clinical picture [26,27,28,29,30,31], anthropometric evaluation may not be an efficient tool for assessing nutritional status. Consistent with previous studies [26,31], in our sample, we reported a nonnormal distribution of weight and height, particularly visible among patients with FAS. Nutritional laboratory markers are an effective and objective tool to assess the nutritional status of children independently of growth restriction associated with PAE [32]. Our study is the first to investigate the nutritional status of children with FASD using laboratory biomarkers of malnutrition.

There are implications that the diet of children with FASD may be deficient in macronutrients. Nguyen et al. established that the protein intake among patients with FASD was significantly lower than in a national sample [10]. However, Werts et al. presented the opposite result, in which the sample protein intake was below the recommended levels in only 7.1% of patients [9]. Similar to Werts et al., Fuglestad et al. reported a protein intake that met dietary recommendations throughout the sample [11]. In our sample, no evidence of hypoproteinemia was found. In fact, a significant number of our patients had increased serum protein and albumin concentration. There are several explanations for this outcome. Relative hyperproteinemia is the result of decreased fluid intake [33]. It has been established that children with FASD have poor satiety as a consequence of their poor impulse control [7,8]. Therefore, relative hypoproteinemia is one of the possible interpretations of the result. However, only an extreme type of malnutrition, such as kwashiorkor disease, can result in severe hypoproteinemia/hypoalbuminemia [34]. In well-studied diseases related to low appetite and decreased caloric intake, such as anorexia nervosa and ARFID, serum albumin and protein concentrations are often normal [35,36] or even elevated compared to controls. This effect is attributed to changes in the distribution between body compartments and increased protein absorption from the gastrointestinal system [35,36,37]. As albumin can easily be transferred between vascular and interstitial spaces, its concentration in serum may also be a consequence of body composition [38,39]. It has been established that the body composition of children with FASD is altered as compared to controls [40,41,42,43]. In selected groups of patients, a relationship between body composition and serum albumin has been identified [44,45,46]. Changes in body composition are another possible explanation for the increase in serum protein/albumin concentration in patients with FASD. However, prealbumin levels were within reference intervals throughout the cohort. As prealbumin is considered the most accurate biomarker of malnutrition regarding protein intake [32,47,48], the general conclusion would be that patients with FASD are not at risk of malnutrition reflected in hypoproteinemia.

Hypercholesterolemia was observed in a significant number of patients. As animal fat overconsumption has not been reported before in this group [9,10,11], this can be considered an unexpected outcome. Susceptibility to high-fat-diet-induced metabolic syndrome associated with PAE was suggested in a study in an animal model of PAE [49]. In a self-report survey, adults with FASD identified metabolic symptoms as one of their main complaints [50]. Researchers also considered PAE being a risk factor for metabolic syndrome [51,52] and liver disease [53,54]. Interestingly, according to the study by Kable et al., no differences were observed between adults with PAE and healthy controls in terms of cholesterol level [55]. Nevertheless, according to the phenomenon called metabolic programming, the adverse intrauterine environment predisposes to a metabolic syndrome in the future [56,57]. PAE is a cause of significant stress for a developing foetus and contributes to the restriction of foetal growth [29,58], which can be considered a significant risk factor for adverse metabolic programming. The hypercholesterolemia observed in a significant proportion of our sample could reflect a predisposition to develop a metabolic syndrome in the future.

Children with FASD are at risk of micronutrient deficiencies, according to previous publications. Authors have underlined deficiencies in vitamin D, E, and K and choline and omega-3-fatty acids [9,10,11]. In our sample, we observed vitamin D deficiency in a significant number of patients, which is consistent with previous findings. However, we identified elevated serum levels of B12 and vitamin E and vitamin A in8%, 7%, and 19% of patients, respectively. Higher levels of vitamin B12 have previously been indicated in children with other neurodevelopmental disorders [59,60]. Diverse intracellular use of vitamin B12 could explain this phenomenon [61]. Essentially, delayed myelination, and neuronal maturation have been described before in FASD, [62,63,64,65,66]. More patients with increased vitamin B12 were identified in the FAS group, although—possibly due to the relatively small sample size—the difference between FAS and ND-PAE was not statistically significant. However, this finding could suggest that a more severe clinical picture (FAS) is associated with elevated vitamin B12 levels due to severely impaired neuronal maturation. Another interpretation links high vitamin B12 levels with oxidative stress. High serum vitamin B12 concentrations have been associated with markers of oxidative stress in cerebrospinal fluid [67]. Oxidative stress is undoubtedly an important pathophysiological pathway leading to cellular damage in FASD [68,69,70,71,72]. Reactive oxygen species (ROS) produced by cytochrome P450 2E1 as a result of ethanol and acetaldehyde metabolism induce cellular apoptosis [73]—this effect has been demonstrated in many FASD models [74,75,76]. A similar explanation could link FASD with increased vitamin E levels. Prenatal supplementation with vitamin E and vitamin A, important antioxidants, is known to alleviate the effect of PAE [77,78,79,80,81]. We hypothesise that vitamin E absorption could be upregulated as a preventive mechanism to reduce oxidative stress. In particular, tocopherol (vitamin E) is one of the most efficient antioxidants [73]. Furthermore, a relationship between cholesterol and vitamin E concentration is well established [82], which is consistent with our findings. Prenatal alcohol consumption is known to affect postnatal intestinal absorption of zinc [83]. Women with a history of alcohol abuse are at risk of zinc deficiency [84,85]. As the nutritional status of the biological mothers of the patients in our cohort is unknown, we can speculate that patients with increased serum zinc concentration could have experienced zinc deficiency during pregnancy.

This is the first study to assess the nutritional status of children with FASD using laboratory biomarkers. The reported findings shed new light on nutrimetabolomics in this group of patients. The relatively large sample size, compared to previous studies that evaluated the matter with dietary reports [9,10,11], can also be considered a strength of our study.

The findings of this report are subject to at least three limitations. First, we excluded children who followed an elimination diet and asked about dietary supplementation, but we did not obtain precise data on daily intake of nutrients in our group. Second, since none of the hospitalised patients (neither acute nor diagnostic admissions) can be considered “healthy” and performing venipuncture and blood sampling only for study purposes from healthy children of all ages can be considered unethical, we resigned from the control group and confronted our results with well-established population norms. Third, we collected information on the type of custody, but we did not include information on the length of the actual caregiver’s custody, which could affect the nutritional status of a child. Notwithstanding the limitations, this study suggests that metabolic aspects of FASD could alter the actual effect of nutrient intake. More nutrimetabolomic research is needed in this group of patients to fill the knowledge gaps.

## 5. Conclusions

In conclusion, this study provides important information on the nutritional status of children with FASD and shows that malnutrition in this population may not be adequately measured using conventional anthropometric techniques. We found no evidence of the expected hypoproteinemia; instead, elevated serum protein and albumin levels were observed, which may be linked to metabolic changes and changed body composition. Unexpectedly, high rates of hypercholesterolemia were also observed in the study, which may indicate a risk of metabolic syndrome. The findings, including elevated vitamin B12 and vitamin E levels, suggest that metabolic disturbances associated with FASD can affect nutrient metabolism, possibly through oxidative stress and impaired neuronal maturation. The identified vitamin D deficit aligns with prior research in this domain. These findings highlight the complexity of metabolism in a group of children with FASD. The work emphasises how crucial it is to continue to conduct research on nutrimetabolomics. Such studies are essential to advance our knowledge of the specific nutritional difficulties this population faces and to support the development of more effective therapeutic care strategies.

## Figures and Tables

**Table 1:** Analytical methods and reference intervals used in the study.

Laboratory Test	Method and Analyzer	Age and Sex Subgroups	Age- and Sex-Specific Reference Intervals	Source of Reference Intervals
Total protein	Biuret, Abbott Architect	1–<6 years	61–75 g/L	Colantonio D.A. et al. [15]
		6–<9 years	64–77 g/L	
		≥9 years, females	65–81 g/L	
Albumin	BCG, Abbott Architect	1–<8 years	38–47 g/L	Colantonio D.A. et al. [15]
		8–<15 years	41–48 g/L	
		≥15 years, females	40–49 g/L	
		≥15 years, males	41–51 g/L	
Prealbumin	Immunoturbidimetric, Abbott Alinity	1–<12 years, females	0.12–0.30 g/L	Laboratory information
		1–<12 years, females	0.11–0.34 g/L	
		≥12 years, females	0.16–0.38 g/L	
		≥12 years, males	0.18–0.45 g/L	
Cholesterol	Enzymatic, Abbott Architect	≥1 year	2.90–5.40 mmol/L	Colantonio D.A. et al. [15]
Ferritin	CMIA, Abbott Architect	1–<5 years	5.3–99.9 ng/dL	Bailey D. et al. [16]
		5–<14 years	13.7–78.8 ng/dL	
		≥14 years, females	5.5–67.4 ng/dL	
		14–<16 years, males	12.7–82.2 ng/dL	
		≥16 years, males	11.1–171.9 ng/dL	
Vitamin B12	CMIA, Abbott Alinity	1–<9 years	209–1190 pmol/L	Bailey D. et al. [16]
		9–<14 years	186–830 pmol/L	
		14–<17 years	180–655 pmol/L	
Vitamin E	HPLC	≥1 year	6–14 mg/L	Raizman E.J. et al. [17]
Vitamin A	HPLC	<11 years	0.275–0.444 mg/L	Raizman E.J. et al. [17]
		11–<16 years	0.249–0.550 mg/L	
		≥13 years	0.287–0.751 mg/L	
Vitamin D	CLIA, Diasorin Liaison XL	All	30–100 ng/mL	Rusińska A. et al. [18]
Parathormone	CMIA, Abbott Architect	1–<9 years	16.2–63.0 ng/mL	Bailey D. et al. [16]
		9–<17 years	21.9–87.6 pg/mL	
Phosphate	Phosphomolybdate, Abbott Architect	1–<5 years	1.38–2.19 mmol/L	Colantonio D.A. et al. [15]
		5–<13 years	1.33–1.92 mmol/L	
		13–<16 years, females	1.02–1.79 mmol/L	
		13–<16 years, males	1.14–1.99 mmol/L	
		≥16 years	0.95–1.62 mmol/L	
Magnesium	Enzymatic, Abbott Architect	1–<4 years, females	0.62–0.90 mmol/L	Soldin P. et al. [19]
		1–<4 years, males	0.65–0.90 mmol/L	
		4–<11 years, females	0.66–1.03 mmol/L	
		4–<11 years, males	0.61–0.90 mmol/L	
		11–<16 years, females	0.66–0.86 mmol/L	
		11–<16 years, males	0.55–0.84 mmol/L	
		≥16 years, females	0.61–0.78 mmol/L	
		≥16 years, males	0.64–0.86 mmol/L	
Total calcium	Arsenazo III, Abbott Architect	≥1 year	2.29–2.63 mmol/L	Colantonio D.A. et al. [15]
Ionised calcium	Potentiometric, Radiometer ABL90 Flex	All	1.19–1.33 mmol/L	Bohn M.K. [20]
Zinc	ICP-MS	<14 years	7.7–15.0 μmol/L	Laboratory information
		≥14 years	9.0–18.0 μmol/L	
Red blood cells	Impedance, Sysmex XN 550	2 months–<15 years	3.8–5.2 × 10^6^/μL	Pelt J.L. et al. [21]
		≥15 years, females	4.0–5.2 × 10^6^/μL	
		≥15 years, males	4.4–5.7 × 10^6^/μL	
Haemoglobin	Sodium lauryl sulphate, Sysmex XN 550	1–<2 years	10.0–13.5 g/dL	
		2–<5 years	10.5–13.5 g/dL	
		5–<10 years	10.9–14.9 g/dL	
		10–<15 years	11.4–15.4 g/dL	
		≥15 years, females	11.8–15.2 g/dL	
		≥15 years, males	13.4–17.0 g/dL	
Haematocrit	Impedance, Sysmex XN 550	6 months–<2 years	33–39%	
		2–<7 years	34–40%	
		7–<13 years	35–45%	
		≥13 years, females	37–46%	
		≥13 years, males	41–50%	
MCV	Calculated, Sysmex XN 550	6 months–<2 years	70.0–90.0 fL	
		2–<5 years	74.0–94.0 fL	
		5–<8 years	76.0–96.0 fL	
		8–<15 years	78.0–98.0 fL	
		≥15 years	82.5–97.4 fL	
White blood cells	Flow cytometry, Sysmex XN 550	6 months–<2 years	6.0–17.5 × 10^3^/μL	
		2–<6 years	5.0–15.5 × 10^3^/μL	
		6–<10 years	4.5–14.5 × 10^3^/μL	
		10–<15 years	4.5–13.5 × 10^3^/μL	
		≥15 years	3.7–9.2 × 10^3^/μL	
Neutrophils	Flow cytometry, Sysmex XN 550	2 weeks–<2 years	1.0–8.5 × 10^3^/μL	
		2–<7 years	1.5–8.0 × 10^3^/μL	
		7–<11 years	1.5–8.5 × 10^3^/μL	
		11–16 years	1.8–8.0 × 10^3^/μL	
Lymphocytes	Flow cytometry, Sysmex XN 550	1–<2 years	1.1–8.6 × 10^3^/μL	
		2–<6 years	1.5–7.0 × 10^3^/μL	
		6–<12 years	0.9–3.4 × 10^3^/μL	
		12–16 years	1.2–5.2 × 10^3^/μL	
Monocytes	Flow cytometry, Sysmex XN 550	6 months–<2 years	0.25–1.15 × 10^3^/μL	
		2–<6 years	0.19–0.94 × 10^3^/μL	
		6–<12 years	0.19–0.85 × 10^3^/μL	
		≥12 years	0.18–0.78 × 10^3^/μL	

Abbreviations: BCG, bromocresol green; CMIA, chemiluminescent microparticle immunoassay; CLIA, chemiluminescence immunoassay; HPLC, high-performance liquid chromatography; ICP-MS, inductively coupled plasma–mass spectrometry; MCV, mean corpuscular volume.

**Table 2:** Demographic and laboratory data of the patients studied. Data are shown as arithmetic mean ± standard deviation or median (lower; upper quartile).

Variable	Whole Cohort (*n =* 75)	FAS (*n =* 25)	ND-PAE (*n =* 50)	*p*-Value
Age, years	7 (3; 9)	8 (3; 9)	6 (4; 9)	0.8
Male sex, *n* (%)	39 (52)	14 (56)	25 (50)	0.6
Institutional care, *n* (%)	3 (4)	0	3 (6)	0.093
Foster family, *n* (%)	34 (45)	10 (40)	24 (48)	
Adoptive family, *n* (%)	34 (44)	15 (60)	18 (36)	
Biological family, *n* (%)	5 (7)	0	5 (10)	
Total protein, g/L	71.8 ± 4.5	72.0 ± 3.9	71.7 ± 4.8	0.7
Albumin, g/L	46.9 ± 2.7	46.7 ± 2.3	47.1 ± 2.9	0.5
Prealbumin, g/L	0.19 (0.17; 0.22)	0.20 (0.18; 0.23)	0.19 (0.17; 0.21)	0.3
Cholesterol, mmol/L	4.36 ± 0.65	4.48 ± 0.63	4.31 ± 0.66	0.3
Ferritin, ng/mL	25.7 (19.1; 37.4)	25.0 (18.6; 39.5)	25.7 (19.3; 34.5)	0.7
Vitamin B12, pg/mL	531 (435; 696)	526 (464; 728)	540 (427; 682)	0.7
Vitamin E, mg/L	12.1 ± 2.7	12.3 ± 2.5	12.0 ± 2.8	0.6
Vitamin A, mg/L	0.33 (0.29; 0.37)	0.34 (0.29; 0.41)	0.32 (0.29; 0.37)	0.2
Vitamin D, ng/mL	34.5 ± 10.7	35.0 ± 7.9	34.3 ± 12.0	0.8
Parathormone, pg/mL	35.2 (29.5; 42.8)	35.4 (30.6; 42.6)	34.9 (29.5; 43.6)	0.8
Phosphate, mmol/L	1.58 ± 0.19	1.61 ± 0.20	1.57 ± 0.19	0.4
Magnesium, mmol/L	0.85 (0.80; 0.88)	0.85 (0.80; 0.88)	0.85 (0.81; 0.88)	0.9
Total calcium, mmol/L	2.46 ± 0.10	2.44 ± 0.09	2.47 ± 0.11	0.2
Ionised calcium, mmol/L	1.26 (1.23; 1.29)	1.25 (1.23; 1.28)	1.26 (1.23; 1.29)	0.3
Zinc, μmol/L	14.0 ± 2.5	14.3 ± 2.6	13.9 ± 2.5	0.5
Red blood cells, ×10^6^/μL	4.71 ± 0.33	4.66 ± 0.32	4.74 ± 0.34	0.4
Haemoglobin, mg/dL	12.8 ± 0.9	12.6 ± 0.9	12.8 ± 0.9	0.4
Haematocrit, %	37.2 ± 2.4	36.6 ± 2.5	37.5 ± 2.3	0.1
MCV, fL	78.6 (76.9; 80.5)	78.4 (76.7; 80.3)	78.6 (77.1; 80.8)	0.4
White blood cells, ×10^3^/μL	7.35 (5.48; 8.53)	6.08 (5.23; 7.94)	7.56 (5.76; 9.06)	0.1
Neutrophils, ×10^3^/μL	3.11 (2.38; 4.00)	3.14 (2.27; 3.60)	3.04 (2.38; 4.14)	0.6
Lymphocytes, ×10^3^/μL	2.80 (2.23; 4.01)	2.67 (2.23; 3.77)	2.85 (2.24; 4.08)	0.7
Monocytes, ×10^3^/μL	0.54 (0.45; 0.67)	0.51 (0.44; 0.62)	0.60 (0.46; 0.71)	0.9

Abbreviations: FAS, fetal alcohol syndrome; ND-PAE, neurodevelopmental disorder with prenatal alcohol exposure; MCV, mean corpuscular volume.

**Table 3:** Percentages of laboratory results below or above the age- and sex-specific reference intervals.

Variable	Whole Cohort (*n =* 75)		FAS (*n =* 25)		ND-PAE (*n =* 50)		*p*-Value
	Below	Above	Below	Above	Below	Above	
Total protein, *n* (%)	0	13 (17)	0	3 (12)	0	10 (22)	0.3
Albumin, *n* (%)	0	23 (31)	0	7 (28)	0	16 (32)	0.6
Prealbumin, *n* (%)	0	0	0	0	0	0	-
Cholesterol, *n* (%)	0	13 (17)	0	5 (20)	0	8 (16)	0.7
Ferritin, *n* (%)	4 (5)	1 (1)	1 (4)	1 (4)	3 (6)	0	0.3
Vitamin B12, *n* (%)	0	6 (8)	0	4 (16)	0	2 (4)	0.071
Vitamin E, *n* (%)	0	14 (19)	0	5 (20)	0	9 (18)	0.7
Vitamin A, *n* (%)	2 (3)	5 (7)	1 (4)	2 (9)	1 (2)	3 (6)	0.8
Vitamin D, *n* (%)	26 (35)	0	6 (24)	0	20 (40)	0	0.2
Parathormone, *n* (%)	0	2 (3)	0	0	0	2 (4)	0.3
Phosphate, *n* (%)	4 (5)	0	2 (8)	0	2 (4)	0	0.5
Magnesium, *n* (%)	0	3 (4)	0	1 (4)	0	2 (4)	1.0
Total calcium, *n* (%)	1 (1)	3 (4)	1 (4)	1 (4)	0	2 (4)	0.4
Ionised calcium, *n* (%)	2 (3)	6 (8)	2 (8)	2 (8)	0	4 (8)	0.1
Zinc, *n* (%)	1 (1)	14 (19)	1 (4)	5 (20)	0	9 (19)	0.4
Red blood cells, *n* (%)	0	4 (5)	0	1 (4)	0	14 (30)	0.7
Haemoglobin, *n* (%)	0	1 (1)	0	0	0	1 (2)	0.5
Haematocrit, *n* (%)	5 (7)	1 (1)	4 (16)	0	1 (2)	1 (2)	0.067
MCV, *n* (%)	10 (13)	3 (4)	4 (16)	0	6 (12)	3 (6)	0.4
White blood cells, *n* (%)	3 (4)	2 (3)	2 (8)	1 (4)	1 (2)	1 (2)	0.4
Neutrophils, *n* (%)	3 (4)	3 (4)	1 (4)	0	2 (4)	3 (6)	0.4
Lymphocytes, *n* (%)	1 (1)	10 (13)	0	2 (8)	1 (2)	8 (16)	0.4
Monocytes, *n* (%)	0	6 (8)	0	2 (8)	0	4 (8)	1.0

Abbreviations: FAS, fetal alcohol syndrome; ND-PAE, neurodevelopmental disorder with prenatal alcohol exposure; MCV, mean corpuscular volume.

**Table 4:** Statistically significant correlations between laboratory results studied and weight, height, and BMI percentiles. R denotes the Spearman correlation coefficient (the percentile categories were treated as an ordinal variable).

	Weight Percentile		Height Percentile		BMI Percentile	
	R	*p*-Value	R	*p*-Value	R	*p*-Value
Prealbumin	0.24	0.039	NS		NS	
Vitamin B12	0.30	0.009	NS		0.30	0.010
Vitamin D	−0.23	0.050	NS		NS	
Zinc	0.24	0.042	0.27	0.021	0.29	0.014
Haemoglobin	0.27	0.022	NS		NS	
Haematocrit	0.23	0.046	NS		NS	
White blood cells	−0.36	0.002	NS		−0.26	0.025
Neutrophils	−0.28	0.015	NS		NS	
Lymphocytes	−0.40	<0.001	NS		−0.34	0.003

Abbreviation: NS, non-significant result; BMI, body mass index.

**Table 5:** Statistically significant associations between demographic data and the studied laboratory test results below or above the age- and sex-specific reference intervals.

Laboratory Test Result	Age			Type of Custody		
	≤2 Years (*n =* 11)	>2 Years (*n =* 64)	*p*-Value	Foster or Institutional (*n =* 37)	Adoptive (*n =* 34)	*p*-Value
Increased albumin, *n* (%)	8 (73)	15 (24)	0.001	17 (46)	3 (9)	<0.001
Increased zinc, *n* (%)	NS			11 (30)	0	0.002

Abbreviations: NS—non significant result.

## Data Availability

The data presented in this study are available on request from the corresponding author due to privacy restrictions.
